# Mitochondrial Function in Muscle Stem Cell Fates

**DOI:** 10.3389/fcell.2020.00480

**Published:** 2020-06-16

**Authors:** Debasmita Bhattacharya, Anthony Scimè

**Affiliations:** Molecular, Cellular and Integrative Physiology, Faculty of Health, York University, Toronto, ON, Canada

**Keywords:** mitochondria, satellite cell fates, metabolism, epigenetics, myogenic stem cells

## Abstract

Mitochondria are crucial organelles that control cellular metabolism through an integrated mechanism of energy generation via oxidative phosphorylation. Apart from this canonical role, it is also integral for ROS production, fatty acid metabolism and epigenetic remodeling. Recently, a role for the mitochondria in effecting stem cell fate decisions has gained considerable interest. This is important for skeletal muscle, which exhibits a remarkable property for regeneration following injury, owing to satellite cells (SCs), the adult myogenic stem cells. Mitochondrial function is associated with maintaining and dictating SC fates, linked to metabolic programming during quiescence, activation, self-renewal, proliferation and differentiation. Notably, mitochondrial adaptation might take place to alter SC fates and function in the presence of different environmental cues. This review dissects the contribution of mitochondria to SC operational outcomes, focusing on how their content, function, dynamics and adaptability work to influence SC fate decisions.

## Introduction

Skeletal muscle constitutes a significant percentage of total body mass and is indispensable to physical movements, maintaining postures and in vital actions (Diaz-Vegas et al., 2019; Hood et al., 2019). It is also a primary peripheral tissue important for utilizing glucose and fatty acid for energy generation essential for the prevention of obesity and type 2 diabetes (Dumont et al., 2015a; Nguyen et al., 2019). It is made up of thousands of long and cylindrical multinucleated muscle fibers surrounded by the sarcolemma, a lipid bi-layer membrane that attaches a complex extracellular matrix, which is in contact with the basement membrane (Dumont et al., 2015a). The fibers are categorized on the basis of various myosin heavy chain isoforms ranging from oxidative to glycolytic fibers (Schiaffino and Reggiani, 2011).

One of the remarkable properties of skeletal muscle is effective regeneration of muscle fibers to maintain their normal physiology. Myofiber turnover is an ongoing process during the lifetime of an individual to maintain proper muscle tissue viability (Yin et al., 2013; Dumont et al., 2015b). This is especially important during aging, diseases such as Duchenne muscular dystrophy and disuse where skeletal muscle fibers are frequently damaged (Yin et al., 2013; Dumont et al., 2015b). Successful regeneration of skeletal muscle is made possible by the adult myogenic stem cell population known as satellite cells (SCs) (Yin et al., 2013). These are located between the basal lamina and muscle fiber sarcolemma, where they are well positioned to receive signals from the surrounding environment (Dumont et al., 2015b). Inefficient muscle regeneration replaces muscle with fibrotic tissues that cause poor contraction and lead to progressive loss of muscle strength as observed during aging or muscle wasting diseases (Mann et al., 2011). Thus, an understanding of the control mechanisms that dictate SC fate decisions is crucial to improve skeletal muscle regeneration potential.

Advances in the stem cell field have unveiled the importance of mitochondria in controlling stem cell behavior including their fate decisions to self-renew or differentiate (Ryall et al., 2015a; Khacho et al., 2016). Mitochondria are central bioenergetic hubs involved in generating ATP via oxidative phosphorylation (Oxphos) that is associated with their dynamic morphological transformations (Chandel, 2018). Energy output is made possible by housing electron transport chain (ETC) complexes that are supplied by reducing agents made by the tricarboxylic acid (TCA) cycle (Chandel, 2018). Apart from this main role, mitochondria also have other functions such as macromolecule synthesis, apoptosis regulation, production of reactive oxygen species (ROS), calcium homeostasis that might regulate various signaling pathways (Chandel, 2018).

Unlike for terminally differentiated myofibers [see reviews, Diaz-Vegas et al. (2019), Hood et al. (2019)], there is a paucity of data for mitochondrial role in SCs. Despite this shortcoming the past few years have witnessed mitochondrial function linked to dictating SC fate determination, whether to maintain quiescence, become activated, self-renew or commit to differentiate. Importantly, mitochondrial energy output is associated with SC functional outcomes (Lyons et al., 2004; Folmes et al., 2012; Ryall et al., 2015b; Hori et al., 2019). Besides harvesting energy, mitochondria also have other essential functions linked to SC fate decisions. Despite having deleterious effects on SCs, ROS produced by leakage of electrons in the ETC are linked to their fate choices (Le Moal et al., 2017). Moreover, several TCA cycle intermediates are known to act as cofactors for different histone and DNA modifying enzymes leading to epigenetic remodeling required for SC self-renewal, commitment and differentiation (Matilainen et al., 2017). Finally, not much is known regarding mitochondria adaptation to different environmental cues that have a profound impact on SC function predisposing myogenic quality. This review will focus on the contribution of mitochondria to SC operational outcomes, focusing on how their content, function, dynamics and adaptability work to influence SC fate decisions.

## Satellite Cell Differentiation

Over half a century has passed since a population of mononucleated cells was discovered between the basal lamina and plasma membrane of skeletal muscle fibers (Katz, 1961; Mauro, 1961) representing 2–10% of the total myonuclei (White et al., 2010). Due to their peripheral localization they were termed satellite cells (SCs). At that time, their close proximity to the myofiber raised the hypothesis that they might be involved in regeneration and growth. A possibility proved correct with advances over many years that showed SCs as the primary myogenic stem cells necessary for the regeneration and maintenance of skeletal muscle fibers.

As with other stem cells, SCs have the ability to self-renew and give rise to functional progeny. Usually SCs are quiescent within their niche and enter the cell cycle when activated by external cues such as injury or trauma. Their ideal positioning enables them to receive local signals from muscle fibers, fibroblasts, endothelial cells and importantly systemic factors from blood vessels with which they co-localize (Yin et al., 2013). After activation some SCs become committed to enter the myogenic lineage as myogenic precursor cells (MPCs), whereas others self-renew to replenish their pool. In turn the MPCs proliferate and exit the cell cycle differentiating into myocytes that repair damaged muscle by either fusing with pre-existing fibers or together to form entirely new fibers (Yin et al., 2013; Dumont et al., 2015b).

Quiescent SCs are characterized by the expression of paired homeobox transcription factor 7 (Pax7) (Seale et al., 2000). They constitute a heterogeneous population, with most representing a committed population that had expressed myogenic factor 5 (Myf5) and a small uncommitted number (10%) that had never expressed Myf5 (Kuang et al., 2007). The later population can undergo asymmetric division producing progeny with and without Myf5 expression. Apart from asymmetric divisions, both SC populations also maintain and expand their population by symmetric division (Kuang et al., 2007; Yin et al., 2013; Dumont et al., 2015b). The equilibrium between symmetric and asymmetric division to maintain the homeostatic population of SCs is preserved by Wingless-type MMTV integration site 7A (Wnt7A) signaling with its receptor Frizzled 7 (Fzd7) (Le Grand et al., 2009). Their signaling pathway dictates the polarity, parallel or perpendicular, of mitotic division with respect to the basal lamina. Wnt7A by binding Fzd7 induces symmetric division by causing the SC to divide in a parallel orientation producing two identical daughter cells. On the other hand, the absence of Wnt7A favors cellular division perpendicular to the basal lamina resulting in asymmetric division. This produces one daughter cell retaining SC characteristics and another daughter cell that is committed to myogenic program.

During the commitment step into MPCs, SCs likewise with Myf5 also express another muscle regulatory factor known as myogenic determinant factor 1 (MyoD). The proliferating MPCs destined for differentiation downregulate Pax7 and up-regulate muscle specific transcription factor myogenin (Zammit et al., 2006; Olguin et al., 2007; Yin et al., 2013). This marks the entry of MPCs into the differentiation phase accompanied by cessation of proliferation. Differentiating MPCs initiate expression of various muscle specific genes encoding structural proteins resulting in their fusion (Przewozniak et al., 2013).

Alternatively, another model for SC activation and commitment suggest that satellite cells have a more homogeneous character, such that their fate choice to self-renew or differentiate occurs from MyoD^+^ progeny. In this case, all activated SCs co-express Pax7 and MyoD wherein most will undergo rapid but limited proliferation, eventually down-regulating Pax7 and up-regulating myogenin as they differentiate (Zammit et al., 2006). Moreover, a few Pax7^+^MyoD^+^ SC progeny will lose MyoD but continue to maintain Pax7 expression. Some of these divide slowly to replenish their pool or they directly differentiate (Zammit et al., 2006). Thus, orchestrated regulation of SC activation, self-renewal, commitment, proliferation and differentiation are necessary for skeletal muscle regeneration, repair and maintenance.

## Mitochondrial Function and Dynamics

Mitochondria are double membranous organelles that consist of outer and inner membranes, the latter forming numerous folds called cristae (Hood et al., 2019). The area between the outer and inner membranes is called the intermembrane space, whereas the matrix is the space encompassed by the inner membrane. Mitochondria are considered bioenergetic hubs where ATP is produced via Oxphos by the ETC, consisting of five distinct complexes which are located on the inner membrane and cristae (Chandel, 2018). Apart from their canonical function of energy production, mitochondria generate diverse functions such as macromolecule synthesis, apoptosis regulation, redox balance, and calcium homeostasis that might regulate various signaling pathways (Chandel, 2018). Notably, mitochondria matrix house the TCA cycle that performs a series of reactions resulting in the formation of reducing equivalents that include reduced nicotinamide adenine dinucleotide (NADH) and reduced flavin adenine dinucleotide (FADH_2_), which are then oxidized by the ETC to drive ATP production (Martinez-Reyes and Chandel, 2020). The TCA cycle can be prompted by pyruvate formed from glycolysis that enters the cycle either as acetyl CoA through the action of pyruvate dehydrogenase (Pdh) or as oxaloacetate via pyruvate carboxylase (Martinez-Reyes and Chandel, 2020). Apart from pyruvate, β-oxidation of fatty acids and glutaminolysis, also drive the production of NADH and FADH_2_ in the mitochondria (Martinez-Reyes and Chandel, 2020). The electrons formed from oxidation of the reducing equivalents migrate through ETC complexes to the final electron acceptor oxygen that is reduced to water. This passing of electrons through ETC is accompanied by pumping of protons from the mitochondrial matrix to the intermembrane space causing an electrochemical gradient in the inner mitochondrial membrane (Martinez-Reyes and Chandel, 2020). This results in flowback of protons into the matrix through complex V (ATP synthase), which produces ATP from ADP (Martinez-Reyes and Chandel, 2020). During this process, ROS might be generated by leakage of electrons in complexes I and III which might cause oxidative stress to cells (Martinez-Reyes and Chandel, 2020). To protect the cells from the potential harmful effect of the ROS, mitochondria have their own antioxidant defense systems (Oyewole and Birch-Machin, 2015). Notable among them are glutathione peroxidases (GPxs) and superoxide dismutase (SOD) which are able to scavenge ROS to maintain cellular homeostasis (Schieber and Chandel, 2014). Although considered harmful, in some stem cell types an optimal level of ROS has been shown to be important for various signaling pathways involved in proliferation, differentiation and physiological adaptation to stress (Schieber and Chandel, 2014). Depending on their efficiency, antioxidants can keep the level of ROS at an optimum level, so that it can act as a signaling molecule (Snezhkina et al., 2019).

Mitochondria harbor double stranded circular DNA (mtDNA) that generates polycistronic transcripts which encode thirteen mitochondrial genes, which are functional components of four out of five ETC complexes, thus limiting and crucial for ATP synthesis (Mitra et al., 2009). This was evident in diseases and aging where mtDNA mutations caused reduction in mitochondrial function and Oxphos (Carelli and Chan, 2014; Ryzhkova et al., 2018). The significance of mtDNA to ATP generation was underscored by experiments using a high throughput screen to assess real time ATP concentration in live human cells. In this case, a deficiency in mitochondrial encoded genes highlighted a decrease in mitochondria ATP generation (Mendelsohn et al., 2018).

The number of mitochondria within a cell is determined by two opposing forces, which are biogenesis and mitophagy that create and eliminate mitochondria, respectively (Hood et al., 2019). Moreover, mitochondria organization help to maintain metabolic homeostasis by controlling the capacity of ATP generation (Khacho and Slack, 2017). The switch in mitochondrial dynamics caused by fusion or fission are connected to differential energy generation capacities (Khacho et al., 2016). Mitochondrial fusion is a two-stage process where the outer membrane is fused by mitofusin I (MfnI) and II (MfnII), followed by fusion of inner membrane by optic atrophy 1 (OpaI). Mitochondrial fusion produces elongated mitochondria that are associated with increased Oxphos activity mainly through its regulation of mtDNA (Mitra et al., 2009; Mishra and Chan, 2016; Song and Hwang, 2018; Silva Ramos et al., 2019). Deterioration of mitochondrial fusion results in a decrease in mtDNA content, ETC function and mitochondrial membrane potential (Chen et al., 2005, 2010). Moreover, both the fusion factors are crucial for ETC complex assembly and function which indicate that elongation of mitochondria is conducive to energy generation (Cogliati et al., 2013; Mourier et al., 2015). Conversely, fission is associated with division of mitochondria by dynamin related protein 1 (Drp1) and fission protein 1 (Fsn1). Fission results in augmented mitochondrial fragmentation that causes increased oxidative stress and reduced ATP production (Jheng et al., 2012).

## Mitochondrial Oxphos and Satellite Cell Fates

**FIGURE 1:**
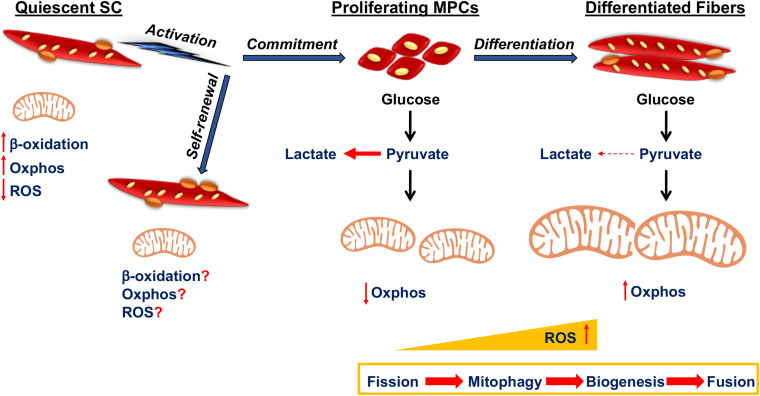
Mitochondrial function in satellite cell fates. Quiescent satellite cells (SCs) utilize fatty acids through β-oxidation for Oxphos in the mitochondria. Activation of quiescent SCs might potentiate their self-renewal or formation of myogenic progenitor cell (MPC) progeny that undergo proliferation and eventually differentiate as part of myofibers. Mitochondrial metabolism is poorly elucidated in self-renewing SCs, whereas MPCs rely on glycolysis to obtain energy for rapid division. As differentiation progresses, there is increased mitochondrial fission, followed by mitophagy, after which the mitochondria is repopulated by mitochondrial biogenesis and fusion. ROS levels increase as MPCs begin to differentiate.

SCs are characterized by dynamic metabolic reprogramming during different stages of the differentiation process from predominantly Oxphos in quiescence to up-regulation of glycolysis during activation and proliferation, back to reliance on Oxphos during terminal differentiation (Figure 1). Quiescent SCs are diminutive with a small layer of cytoplasm surrounding their nuclei. They also have a very few mitochondria, tightly packed around the nucleus, reduced levels of mtDNA and a very low metabolic rate (Latil et al., 2012). This is similar to other adult stem cell populations, such as quiescent long-term hematopoietic stem cells that reside in the bone marrow. These are also characterized by mitochondria with a paucity of mtDNA, reduced mass and poor development (Simsek et al., 2010). However, unlike long-term hematopoietic stem cells, quiescent SCs barely depend on glycolysis and rely more on the mitochondria to produce ATP through β-oxidation of fatty acids and Oxphos (Ryall et al., 2015b). Quiescent SCs are thought to consist of two distinct populations based on differences in mitochondrial density, which are inversely associated with Pax7 levels (Rocheteau et al., 2012). The SC population with low levels of Pax7 had more mitochondria and mtDNA with greater ATP generation compared to the other resident SCs and expressed higher levels of myogenic commitment markers. Moreover, the quiescent SCs with reduced mitochondrial density and lower mitochondrial activity showed increased markers of stemness and decreased markers of myogenic commitment. This population utilized more time before entering the cell cycle for the first round of division after activation, suggesting that they represented a self-renewal population (Rocheteau et al., 2012).

When they become activated, SCs exhibit a metabolic switch from fatty acid oxidation to higher rates of glycolysis (Ryall et al., 2015b). In muscle regeneration experiments, activated SCs isolated from skeletal muscle had a high level of glycolysis and oxygen consumption rate following 3 days of injury (Pala et al., 2018). The reliance on glycolysis provides the proliferating MPCs with macromolecules to meet their anabolic demands during proliferation and might also act to protect them from ROS produced by mitochondrial Oxphos (Folmes et al., 2012). This is evident in other highly proliferative stem cell types. For the highly proliferative undifferentiated embryonic stem cells, self-renewal is maintained by utilizing a high rate of energy generation and a ready supply of macromolecules for cell division, by relying more on glycolysis producing lactate from pyruvate rather than acetyl CoA for Oxphos (Cliff and Dalton, 2017). Recently, single cell RNA sequencing revealed that expression for mitochondrial genes also increased substantially in activated SCs after injury and in committed MPCs that had been isolated from mouse skeletal muscle and cultured *in vitro* (Dell’Orso et al., 2019). This was also associated with a progressive increase in transcripts associated with TCA cycle, ETC complexes and fatty acid synthesis (Dell’Orso et al., 2019).

To increase their proliferative rate, committed SCs have devised several methods to actively reduce Oxphos production in favor of glycolysis. MPC proliferation is associated with hypoxia inducible factor 1α (Hif1α) activation that promotes glycolysis producing lactate, thereby attenuating Oxphos capacity, which is required for differentiation. Indeed, SCs obtained from genetically deleted Hif1α and Hif2α mice had reduced self-renewal and MPCs underwent precocious differentiation (Yang et al., 2017). When MPCs were pre-conditioned in hypoxic environment and transplanted into *mdx* mice, a mouse model for muscle wasting disease, there was improved regeneration capacity of the pre-conditioned MPCs (Liu et al., 2012). Oppositely, Hif1α has also been shown to inhibit differentiation of MPCs (Majmundar et al., 2015). This was mediated through Hif1α mediated inhibition of Wnt signaling that repressed MPC differentiation (Majmundar et al., 2015). Thus, the timing of Hif1α activity might be important for SC and MPC fate decisions (Majmundar et al., 2015).

Another potential mechanism that might downregulate Oxphos capacity in favor of glycolysis in MPCs is activation of pyruvate dehydrogenase kinase (Pdk). In the mitochondria Pdk suppresses Pdh that converts pyruvate to acetyl CoA for the TCA cycle, thereby inhibiting mitochondrial oxidative capacity (Zhang et al., 2014). Pluripotent and some adult stem cells have devised this method to inhibit mitochondrial Oxphos and enhance glycolysis to sustain rapid proliferation (Folmes et al., 2011; Takubo et al., 2013). Although not much is known about regulation of MPCs by Pdk, it was shown that in hypoxia or glucose deprivation, MPCs have higher Pdk activity to support enhanced glycolysis (Abbot et al., 2005; Hori et al., 2019).

Reduced Oxphos capacity in human MPCs during proliferation is maintained by low levels of ETC complex III, IV and V subunits, mitochondrial proteins and enzymes compared to differentiating cells (Hoffmann et al., 2018). A similar profile is observed in proliferating pluripotent stem cells, which are characterized by decreased complex I and complex IV ETC nuclear encoded genes and a reduction in mitochondrial biogenesis regulators such as peroxisome proliferator-activated receptor co-activator 1 beta (*Pgc1*β) and estrogen-related receptor gamma (*ERR*γ) (Zhou et al., 2012; Sperber et al., 2015).

As MPCs begin to differentiate, glycolysis subsides in favor of Oxphos, which is essential for terminal differentiation. Recently, a study showed that conditional deletion of Pdh in SCs that resulted in defective MPC proliferation, also caused poor terminal differentiation and inefficient skeletal muscle regeneration upon injury, suggesting the requirement of pyruvate for efficient Oxphos during differentiation (Hori et al., 2019). Indeed, chloramphenicol, an inhibitor of mitochondrial protein synthesis and function, has been shown to inhibit myogenic differentiation (Rochard et al., 2000; Seyer et al., 2006, 2011). Compared to MPCs, differentiated myofibers have a higher mitochondrial mass composed of pronounced levels of mtDNA, ETC complex proteins and TCA cycle enzymes (Lyons et al., 2004; Kraft et al., 2006; Remels et al., 2010; Hoffmann et al., 2018). The terminally differentiated myofibers require functional mitochondria to sustain the high energy demand of skeletal muscles. Impaired mitochondrial function due to mtDNA mutation have been found to cause many diseases of different organs that include skeletal muscle (Chinnery, 2015).

Energy output from mitochondria during MPC differentiation is associated with their dynamic reorganization by fission, mitophagy, biogenesis and fusion. In metabolic complications such as type 2 diabetes, decreased myogenic differentiation is attributed to impaired mitophagy, which suggests the importance of mitochondrial clearance in facilitating differentiation (Henriksen et al., 2019). Importantly, during early myogenic differentiation, there is upregulation of the fission protein Drp1 mediated mitochondrial fragmentation and subsequent mitophagy by sequestosome 1 (Sin et al., 2016). Drp1 inhibition caused a reduction in differentiation of MPCs with reduced mitochondrial elongation, mtDNA content and mitochondrial biogenesis (Kim et al., 2013). However, if Drp1 is not repressed at later stages, myogenic differentiation does not proceed (De Palma et al., 2010).

Following mitophagy, the mitochondrial biogenesis activator, peroxisome proliferator-activated receptor co-activator 1 alpha (Pgc1α) and the fusion protein Mfn2 rebound mitochondrial dynamics. As differentiation progresses, Opa1 mediates mitochondrial fusion and Pgc1α amplifies mitochondrial biogenesis that together form the dense elongated mitochondrial network pertaining to increased Oxphos reliance in differentiated myotubes (Sin et al., 2016). Ectopic overexpression of Pgc1α in an MPC cell line increased mtDNA, mitochondrial encoded *cytochrome c oxidase* gene expression that enhanced mitochondrial respiration and function (Wu et al., 1999; Baldelli et al., 2014). Importantly, overexpressing Pgc1α in human MPCs boosted myofiber formation capacity both *in vitro* and *in vivo* with enhanced metabolic activity (Haralampieva et al., 2017). A key phenotype of Pgc1α genetically deleted mice is a shift from the oxidative type I and IIa toward the glycolytic type IIx and IIb muscle fibers (Handschin et al., 2007). Interestingly, as this is a whole body Pgc1α deficit, it is not clear if SCs had a role in defining the glycolytic phenotype. Nonetheless, oxidative type fibers contain more SCs than the glycolytic fibers (Gibson and Schultz, 1982) that might be due to Pgc1α mediated increase in SC number as well as proliferation (Dinulovic et al., 2016).

Intriguingly, MyoD has recently been shown to control mitochondrial function that might be necessary for efficient differentiation. By genome wide ChIP seq analysis of a MPC line, it was found that the MyoD interacted with several metabolic genes including those involved in mitochondrial biogenesis, fatty acid oxidation and ETC function (Shintaku et al., 2016). Furthermore, MyoD knockdown reduced TCA cycle activity and β-oxidation of fatty acids (Shintaku et al., 2016).

## Mitochondrial Oxidative Stress and Satellite Cell Fate

In some adult stem cells, ROS signaling is important for fate decisions. For example, in long-term quiescent HSCs barely any ROS is produced, which protects them from precocious differentiation, which is contrary to activated HSCs that require ROS for successful differentiation (Tothova et al., 2007; Owusu-Ansah and Banerjee, 2009; Simsek et al., 2010). For SCs, ROS generation is associated with SC activation and subsequent differentiation, though a functional role for ROS as a signaling factor requires more investigation (Kozakowska et al., 2015; Le Moal et al., 2017). Furthermore, though the contribution of mitochondrial ROS is substantial, it does not preclude the potential ROS effect from other cellular sources that might affect SC fate (Acharya et al., 2013; Le Moal et al., 2017). Despite a reliance on fatty acid metabolism and Oxphos, quiescent SCs have low ROS levels and transcriptomic analysis revealed that quiescent SCs express a greater quantity of antioxidants to protect them from potential harmful effects of ROS (Pallafacchina et al., 2010). SCs obtained from genetically deleted antioxidant superoxide dismutase mice showed lower differentiation ability (Manzano et al., 2013). Indeed, quiescent SCs had a better survival rate than their activated counterparts after hydrogen peroxide treatment, which causes accumulation of cellular generated ROS (Pallafacchina et al., 2010).

Both mitochondrial and nicotinamide adenine dinucleotide phosphate (NADPH) oxidase induced ROS are increased during the MPC differentiation process (Acharya et al., 2013). NADPH oxidase is thought to instigate more mitochondrial ROS by opening of the mitochondria ATP sensitive potassium channels. This allows the surge of potassium ions in the mitochondrial matrix, thereby reducing the mitochondrial membrane potential (Zhang et al., 2001). Attenuated mitochondrial ROS production by inhibiting NADPH oxidase activity, prevented their dysfunction (Doughan et al., 2008). Although important, excessive ROS is detrimental to MPCs by targeting mtDNA and mitochondrial function (Sestili et al., 2009; Sandiford et al., 2014) that leads to swelling and disruption of mitochondria (Sestili et al., 2009). Moreover, deletion of mitochondrial antioxidant, GPx, in MPCs resulted in lower proliferation and differentiation potential (Lee et al., 2006) and MPCs obtained from GPx null mice poorly differentiated with impaired myotube formation (Lee et al., 2006). Also, upregulation of superoxide dismutase in MPCs increased myotube formation (Hidalgo et al., 2014).

Mechanistically, excessive ROS in MPCs is thought to increase nuclear factor kappa beta (NF-κB) (Ardite et al., 2004; Catani et al., 2004), which reduces MyoD levels, thereby inhibiting differentiation (Guttridge et al., 2000; Sandiford et al., 2014). Also, NF-κB mediated activation of YY1, which is a myogenic transcriptional repressor might be another target of ROS mediated inhibition of myogenic differentiation in MPCs (Wang et al., 2007). Importantly, muscle restricted inhibition of NF-κB in mice enhanced SC activation and muscle regeneration (Mourkioti et al., 2006).

The inhibitory function of NF-κB is thought to occur through its classical signaling pathway, which operates via the p65/p50 heterodimer mediated transcription of target genes (Bakkar and Guttridge, 2010). However, contrary to its role as a negative regulator in undifferentiated MPCs, NF-κB also supports differentiating MPCs. This role is achieved by the use of its alternative signaling pathway, which relies on RelB/p52 mediated transcription (Bakkar et al., 2008; Dahlman et al., 2010). In this case, NF-κB mediates transcription of genes that are known to stimulate Oxphos and mitochondrial biogenesis, which are required for myogenic differentiation. Some reports have shown that NF-κB can also promote myogenic differentiation through insulin like growth factor II or p38 Map kinase, which are both known regulators of myogenic differentiation (Conejo et al., 2001; Baeza-Raja and Munoz-Canoves, 2004; Bakkar et al., 2008; Ji et al., 2010). Interestingly, ROS was required by MPCs to exit cell cycle and initiate the process of differentiation by activating p38α Map kinase (L’Honore et al., 2018). This is similar to neural stem cells where ROS has been shown to act as a signaling molecule to activate a cascade of events that upregulate the transcriptional genes for differentiation (Khacho et al., 2016). Inhibition of ROS by the antioxidant n-acetyl-cysteine or of p38α MAP kinase prevented muscle differentiation, but increased the SC pool, suggesting the importance of both the factors in mediating SC differentiation (Richards et al., 2011; Brien et al., 2013). Thus, the negative and positive effects of ROS on SC function might relate to dose and time dependent considerations. An evaluation of other potential factors that influence ROS during SC engagement is important to unravel its bi-faceted role.

Reactive nitrogen species (RNS) might be made from the superoxide leakage in mitochondria and reactive nitric oxide (NO⋅) produced from L-arginine by nitric oxide synthase (Le Moal et al., 2017). NO⋅ is important for SC activation, self-renewal and MPC differentiation (Tatsumi et al., 2006; De Palma et al., 2010; Buono et al., 2012; Rigamonti et al., 2013). NO⋅ might be critical for mitochondrial elongation, required for myogenic differentiation. In primary MPCs, inhibition of nitric oxide synthesis, prevented mitochondrial elongation and myogenic differentiation (De Palma et al., 2010).

## Environmental Influence on Satellite Cell Mitochondrial Adaptation

**FIGURE 2:**
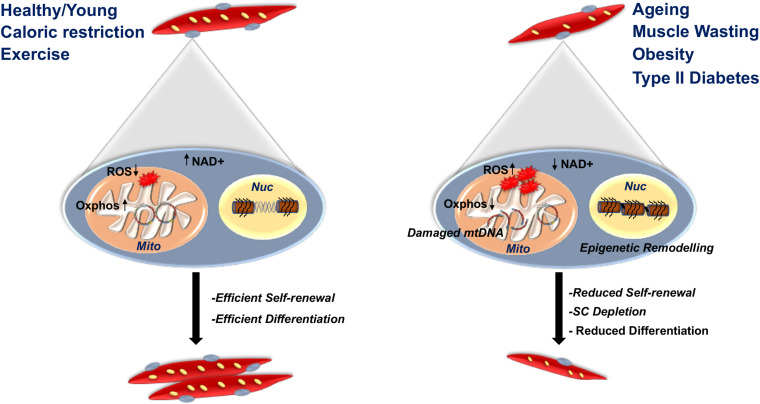
Influence of the micro-environment on mitochondria of quiescent satellite cells. In a healthy, calorie restricted or exercise adapted micro niche, mitochondria from quiescent satellite cells (SCs) operate efficiently, producing Oxphos with very low ROS. This maintains quiescence and effective self-renewal and differentiation. However, mitochondrial adaptations due to metabolic complications as observed in aging, muscle wasting diseases, obesity and type 2 diabetes, lead to fragmented mtDNA, impairment of Oxphos producing capacity, epigenetic alterations and overproduction of ROS that reduce SC self-renewal and differentiation capacity.

Many studies have showed that skeletal muscle adaptation through modifiable physiological perturbations can in part be attributed to innate changes that occurred in the quiescent SC pool (Al-Khalili et al., 2003, 2014; Bell et al., 2010; Consitt et al., 2010; Boyle et al., 2012; Bourlier et al., 2013; Bollinger et al., 2015; Maples et al., 2015; Lund et al., 2017). Thus, reflecting a genetic change in specific DNA sequences and/or from changes to the epigenome in quiescent SCs that would influence the transcriptome and the pathways they influence. Caloric availability is one aspect of the external environment that affects mitochondrial function in SCs (Figure 2). Caloric restricted post-mortem human and mouse SCs have a remarkable ability to stay dormant owing to their anoxic environment with the ability to become activated up to 17 and 14 days, respectively, post death. They are characterized by reduced mitochondrial mass and density similar to one of the two quiescent SC subpopulations that showed increased stem cell markers and reduced commitment (Latil et al., 2012; Rocheteau et al., 2012). Contrarily, despite a lack of nutrients as found in post mortem SCs, those from caloric restricted mice have a higher mitochondrial activity and Oxphos capacity (Cerutti et al., 2014). Importantly, metformin, a caloric restriction mimicking drug that increases Oxphos, maintained SC quiescence *in vitro* and *in vivo* (Pavlidou et al., 2017). SCs obtained from caloric restricted mice had increased nicotinamide adenine dinucleotide (NAD^+^) dependent deacetylase Sirtuin 1 (Sirt1), which had been found to inhibit MPC differentiation and was associated with SC self-renewal (Fulco et al., 2008; Ryall et al., 2015b).

The age-related micro-environment also influences quiescent SCs, as exemplified by their lower numbers and reduced activation potential (Chakkalakal et al., 2012) (Figure 2). Among several contributing factors, an alteration in mitochondrial function associated with aging might influence the decreased number and regeneration potential of SCs (Garcia-Prat et al., 2016a; Pala et al., 2018). Importantly, aging is correlated with reduced mitochondrial content, mtDNA and ATP production (Short et al., 2005). SCs obtained from aged mice showed enhanced ROS mediated irreversible senescence owing to their inability to remove damaged organelles that also include mitochondria (Cosgrove et al., 2014; Garcia-Prat et al., 2016b). In addition, SCs from aged human skeletal muscle displayed decreased expression of genes required for ETC function (Bortoli et al., 2005). Similarly, primary MPCs obtained from the aged skeletal muscles showed impaired mitochondrial Oxphos that attenuated myogenic differentiation (Paasuke et al., 2016).

Severe and progressive skeletal muscle degeneration observed in muscle wasting diseases and complications such as muscular dystrophies and sepsis are also thought to influence SC mitochondria function. Impaired mitochondrial function was revealed in dystrophic mouse models including, the muscular dystrophy (*mdx)* and the double knockout dystrophin strains (Pant et al., 2015; Vila et al., 2017). In addition, absence of dystrophin in SCs reduced the commitment of MPCs by reducing asymmetric division which is thought to exacerbate impaired regeneration in muscular dystrophies (Dumont et al., 2015c; Chang et al., 2018). MPCs obtained from dystrophic mice displayed attenuated oxygen consumption, mitochondrial membrane potential and elevated ROS (Onopiuk et al., 2009). The importance of dystrophin to mitochondria and MPC function was highlighted by restoration of dystrophin in *mdx* MPCs (Matre et al., 2019). This enhanced Oxphos potential to near normal levels, which was associated with improved MPC proliferation and differentiation. Furthermore, transplantation of these intrinsically modified MPCs in dystrophic mice reinstated their regenerative ability (Matre et al., 2019).

Health complications due to sepsis, which might cause long term myopathy is also associated with reduced SC activation, proliferation and regenerative capacity (Rocheteau et al., 2015). Skeletal muscle that had confronted sepsis was characterized by reduced SC numbers, concomitant with defective mitochondria, degraded mtDNA, and increased antioxidant levels to combat excessive ROS (Rocheteau et al., 2015). Normally, on activation, SCs switch from Oxphos to glycolysis that provides macromolecules required for proliferation. However, in sepsis, SC activation was associated with a higher level of Oxphos that was not conducive for division, hence limiting their regenerative capacity (Rocheteau et al., 2015).

Metabolic complications as observed in obesity and type 2 diabetes are also accredited to dysfunctional mitochondria (Figure 2). Notably, many reports have showed that cultured MPCs from obese individuals differentiate into myotubes retaining the metabolic character evident in their skeletal muscles of origin. Biopsy derived SCs obtained from musculus vastus lateralis of exercised human subjects committed and differentiated into myotubes with the same enhanced metabolic benefits that were provided *in vivo* by exercise (Lund et al., 2017). Importantly, SCs cultured *in vitro* to form myotubes had enhanced glucose and lipid oxidation, which suggests that the external cue of exercise is capable of altering mitochondrial function directed by changes in quiescent SCs (Lund et al., 2017). If SCs obtained from lean and obese human subjects were cultured, the latter showed reduced mtDNA copy number and function and altered metabolic derangements (Boyle et al., 2012; Bollinger et al., 2015). Furthermore, the metabolic improvements to skeletal muscle of obese subjects after an exercise training program were also manifested *in vitro*. Indeed, in response to exercise training, cultured primary myotubes from obese donors were characterized by enhanced glucose metabolism (Bourlier et al., 2013).

Similar to obesity, SCs from streptozotocin induced diabetic mice showed impaired myotube formation and regeneration following cardiotoxin induced muscle damage (Jeong et al., 2013). SCs obtained from the mouse model of obesity and diabetes (ob/ob and db/db) showed impaired MPC proliferation and differentiation (Nguyen et al., 2011). In addition, cultured myotubes from diabetic human skeletal muscle showed mitochondrial dysfunction with reduced mitochondrial content and Oxphos capacity (Minet and Gaster, 2011). Moreover, the SCs from human diabetic skeletal muscles lack metabolic flexibility to adapt to the micro-environment (Aguer et al., 2011). When grown in galactose media, which is known to induce oxidative metabolism, SCs from healthy human skeletal muscles acclimatized by enhancing their mitochondrial content and Oxphos capacity, unlike the SCs from diabetic subjects, suggesting that they are obstinate to the changed micro-environment (Aguer et al., 2011).

Intriguingly, environmental cues that can potentially influence mitochondrial function in SCs are also highlighted by a change in Oxphos capacity of uninjured muscle that is distant from a site of a muscle injury (Rodgers et al., 2014). Despite originating from uninjured muscle these SCs had higher mitochondrial activity, mtDNA content, transcriptional activity and regeneration potential compared to SCs from a mouse where no muscle injury was induced. Moreover, the SCs obtained from the uninjured muscle where a muscle injury was generated at a distant site were more primed to become activated (Rodgers et al., 2014). The factor(s) that cause the environmental changes to enhance this phenomenon are unknown.

## Mitochondrial Influence on Epigenetics of SC Fates

**FIGURE 3:**
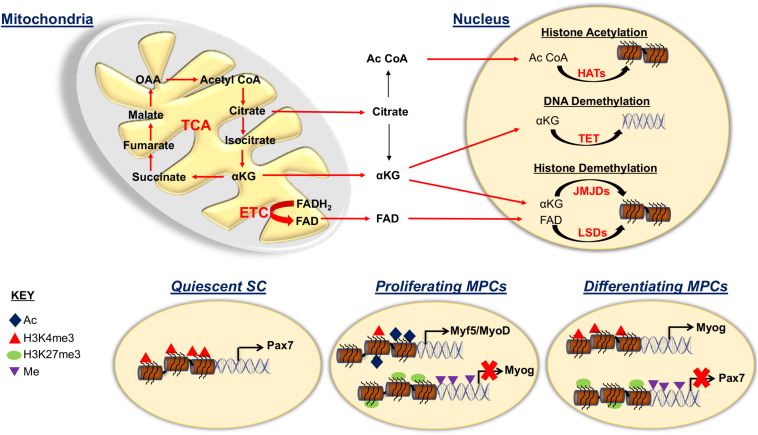
Mitochondrial role for epigenetic regulation of satellite cell fates. Representation illustrating the involvement of the mitochondria for histone acetylation (Ac) and methylation and of DNA methylation (Me) in the maintenance and progression of SC fates. The tricarboxylic acid (TCA) cycle intermediates citrate, acetyl CoA (Ac CoA) and α ketoglutarate (αKG) as well as FAD oxidized from FADH_2_ in the electron transport chain (ETC), are exported from the mitochondria to be used for epigenetic modification of DNA and histones. Acetyl CoA is a substrate of histone acetyltransferases (HATs), αKG is a cofactor for both histone demethylases; Jumonji C domain demethylases (JMJDs) and DNA demethylases ten eleven translocases (TETs) and FAD is a cofactor for lysine specific demethylases (LSDs) that are associated with active transcription. During quiescence, SCs exhibit activated Pax7 due to H3K4me3 modification on the Pax7 promoter. Proliferative myogenic precursor cells (MPCs) contain the repressive histone modification H3K27me3 and DNA methylation at muscle specific gene promoters such as myogenin (Myog). MPCs also contain activation associated histone modifications characterized by augmented acetylation and H3K4me3 on the Myf5 and MyoD promoters. During differentiation, activation and repression associated histone modifications take place for Myog and Pax7 promoters, respectively.

Mitochondria is a source of metabolic intermediates which controls various key enzymes and key metabolites involved in epigenetic regulation (Figure 3) (Matilainen et al., 2017). A crucial facet of epigenetics is histone acetylation, characterized by a dynamic process regulated by opposing effects of enzyme families histone acetyltransferases (HATs) and histone deacetylases (HDACs) (Dutta et al., 2016). Active transcription is associated with HATs that utilize acetyl CoA to transfer acetyl to lysine residues on histones. The HDACs remove the acetyl group from the lysine residues inducing chromatin condensation, which is associated with suppression of gene expression. The availability of acetyl CoA sourced from the mitochondria is crucial to maintain histone acetylation and is strictly dependent on the energy status of the cell (Wellen et al., 2009; Evertts et al., 2013). In the TCA cycle, acetyl CoA and oxaloacetate form citrate that must be exported to the cytoplasm to be reconverted into acetyl CoA by utilizing ATP citrate lyase. During glycolytic energy production, as is the case in activated SCs and proliferating MPCs, high levels of acetyl CoA is produced that leads to histone acetylation (Yucel et al., 2019). A similar phenomenon is observed in self-renewing pluripotent stem cells which have a high rate of glycolysis coupled to high levels of histone acetylation (Xie et al., 2009; Tan et al., 2013).

The HAT, p300 is essential for MyoD activation and its knockdown impaired MPC differentiation (Roth et al., 2003; Fauquier et al., 2018). Furthermore, *MyoD* gene expression is prevented by the myogenic transcriptional repressor Snail1/2 along with histone deacetylases (HDACs) that keep the chromatin in hypoacetylated states, thereby preventing differentiation (Soleimani et al., 2012; Cho et al., 2015). Snail1/2 null myoblasts showed precocious differentiation due to the absence of repression on *MyoD* promoter (Soleimani et al., 2012). This regulatory paradigm that controls the switch in *MyoD* gene expression is important to maintain MPC proliferation and direct differentiation. In contrast to this, in low metabolic states, as with quiescent SCs, acetyl CoA is less available, contributing to less histone acetylation (Yucel et al., 2019). During MPC differentiation, glycolysis derived acetyl CoA is utilized by the TCA cycle to produce more reducing agents because of the elevated energy demand, thus decreasing the availability of acetyl CoA for histone acetylation (Moussaieff et al., 2015; Yucel et al., 2019).

Moreover, in SCs, the energy status determined by the level of NAD^+^/NADH connects the metabolic status of the cells to epigenetic modification. In quiescent SCs, where glycolysis is negligible, high NAD^+^/NADH activates Sirt1, which mediates deacetylation of histones, in particular reduced acetylation of H4K16. However, activated SCs associated with glycolysis decreases NAD^+^/NADH levels, resulting in reduced Sirt1 engagement, which enhances acetylation that increases transcriptional activation of myogenic regulatory factors (Ryall et al., 2015b).

Histone methylation can either repress or activate gene expression by modifying a single lysine or arginine residue on histone proteins (Ducker and Rabinowitz, 2017). The enzymes responsible for methyl group transfer are histone methyl transferases (HMTs) that add a methyl group and demethylases (HDMs) that remove it. Mitochondrial function is crucial for histone methylation, as both HMTs and HDMs require S-adenosyl methionine (SAM) which is produced by the reaction between methionine and ATP (Teperino et al., 2010). Also, the lysine specific demethylase (LSD) is dependent on flavin adenine dinucleotide (FAD), a cofactor of TCA cycle, and Jumonji C domain demethylase (JMJD) is regulated by the TCA cycle intermediate α ketoglutarate (Teperino et al., 2010). Global and site specific histone methylation has revealed that quiescent SCs have high levels of H3K4me3 associated with active transcription of Pax7 and undetectable level of H3K27me3 (Liu et al., 2013; Machado et al., 2017). Activated SCs have the repressive mark, H3K27me3, on the myogenin promoter, thought to prevent premature differentiation (Juan et al., 2011; Liu et al., 2013). Intriguingly, a potential cause of functional decline with aging is linked to an accumulation of H3K27me3 in quiescent SCs (Liu et al., 2013). Moreover, cultured SCs from type 2 diabetic and obese human skeletal muscle also showed increased H3K27me3 histone marks that was correlated with dysregulated muscle differentiation (Varemo et al., 2017).

Histone methyl transferase Suv4-20H1 maintains SC quiescence by recruiting the repressive marker H4K20me2 on the MyoD promoter, thereby preventing its activation (Boonsanay et al., 2016). When muscle stem cells differentiate, there is an upregulation of H3K27me3 near promoters of cell cycle genes and H3K4me3 on the myogenin promoter (Blais and Dynlacht, 2007; McKinnell et al., 2008; Sebastian et al., 2009). In addition, there is an increase of the demethylase LSD activity on histones near myogenic transcription factors during differentiation (Choi et al., 2014, 2015; Scionti et al., 2017). Indeed, LSD genetically deleted MPCs and mice were impaired for differentiation and regeneration capacity, respectively (Choi et al., 2010; Tosic et al., 2018). Also, isoforms of JMJD, DN-JMJD2A and JMJD2C are recruited to Myogenin and MyoD promoters respectively, which help to propagate MPC differentiation by removing the repressive histone marks from the promoter (Verrier et al., 2011; Jung et al., 2015). In parallel to this, the activity of the key lysine methyltransferases such as G9a and enhancer of zeste homolog 2 (Ezh2), which are myogenic transcriptional repressors are dampened to propel the differentiation process (Asp et al., 2011; Ling et al., 2012).

DNA methylation generally is linked to silencing genes by the DNA methyltransferase (DNMT) enzymes that methylate cytosine on promoters at the 5′ position, whereas its demethylation occurs by ten eleven translocase (TET) enzymes. Deregulation of TCA cycle affects DNA methylation, indicating the importance of mitochondria in this process (Pastor et al., 2013). Similar to JMJDs, α ketoglutarate is required for TET activity. DNA methylation has been shown to be decreased when MPCs differentiate evidenced by increased demethylation on the myogenin promoter (Palacios et al., 2010). Additionally, DNA methylation prevented *MyoD* gene expression thereby preventing muscle stem cell commitment and 5-aza-2′-deoxycytidine, an anti-demethylating agent, promoted MPC differentiation (Montesano et al., 2013; Laker and Ryall, 2016). Knockdown of TETs lead to a decrease in muscle specific differentiation factors in MPCs (Zhong et al., 2017). Importantly, an altered micro niche associated with aging might deregulate myogenic differentiation through an increase in DNA methylation (Bigot et al., 2015). Similarly, analysis of genome wide DNA methylation during differentiation of MPCs obtained from obese human subjects showed more methylation compared to non-obese subjects that were associated with increased DNMT expression (Davegardh et al., 2017).

## Perspectives

Widely disparate patterns of mitochondria function are thought to influence SC fates including self-renewal, commitment and differentiation. However, many questions remain unanswered. When activated, the quiescent SCs, which rely on the mitochondria for fatty acid oxidation, switch to glycolysis that is necessary for cell division. Though cell division is required for both self-renewal and commitment, the potential role for mitochondrial function, specifically fatty acid oxidation and Oxphos, in symmetrical versus asymmetrical division has not been elucidated. Other issues that remain unresolved pertain to retrograde signaling from the mitochondria to the nucleus that affect SC function. Crucially, epigenetics regulates chromatin structure that is needed to orchestrate SC quiescence and differentiation. Though it is known that TCA cycle metabolites control epigenetic reprogramming, a paucity of data exits for the pathways that coordinate the specific changes in SC mitochondria. Moreover, the exact role for mitochondrial generated ROS on SC nuclear regulation remains obscure, unlike other adult stem cells where downstream mechanisms have been discovered. Indeed, adipogenic differentiation of human adult mesenchymal stem cells is coupled to mitochondrial ROS production that controls the mammalian target of rapamycin (mTorc) pathway that activates peroxisome proliferator-activated receptor gamma (Pparγ) the master regulator of adipocyte differentiation (Tormos et al., 2011). It will be of interest to discover the downstream pathways and genes that might be directly impacted by mitochondrial ROS in SCs.

Finally, an overlooked approach to enhance muscle wellbeing is to increase the pre-existing population of inactivated SCs for a greater innate capacity to augment self-renewal and muscle formation. In this case, there is data lacking for how the mitochondria might be manipulated. One approach would be to understand how environmental stressors modify quiescent SCs at the level of global gene expression and epigenetic changes that had occurred during their adaptation. In this way we can learn how the mitochondria are influenced by pathways and genes in quiescent SCs, which will regulate their metabolic behavior in the future. To this end, single cell RNA and ChIP seq on quiescent SCs under different environmental stressors are required to find out a potential effect on mitochondrial and SC adaptation. Further studies, dissecting the consequence of the altered mitochondrial activity on SC fate associated with metabolic diseases is critical. This will further help in ameliorating decreased regeneration efficacy in aging and other muscle wasting diseases.

## Summary

The transformative events on mitochondrial structure, number and activity in SCs, the predominant myogenic stem cell, shape how muscle regeneration will proceed. Moreover, mitochondrial function and dynamics wrought through environmental cues affect Oxphos, ROS production and TCA cycle intermediates that directly impact SC plasticity and differentiation. Thus, evaluation of mitochondrial function in SCs will provide new avenues to target for improvements against myogenic impairments and diseases.

## Author Contributions

DB and AS contributed to the conception, design, acquisition, analysis, interpretation of the work, drafting, illustrating, and revising the work.

## Conflict of Interest

The authors declare that the research was conducted in the absence of any commercial or financial relationships that could be construed as a potential conflict of interest.
